# Natural Agents Modulating Ferroptosis in Cancer: Molecular Pathways and Therapeutic Perspectives

**DOI:** 10.1111/jcmm.70834

**Published:** 2025-09-07

**Authors:** Md. Al Amin, Mehrukh Zehravi, Sherouk Hussein Sweilam, Patibandla Jahnavi, Jeetendra Kumar Gupta, Varikalla Rajashakar, Rajeshwar Vodeti, Abdul Ajeed Mohathasim Billah, G. Dharmamoorthy, Uppuluri Varuna Naga Venkata Arjun, Voleti Vijaya Kumar, Muath Suliman, Talha Bin Emran

**Affiliations:** ^1^ Department of Pharmacy, Faculty of Health and Life Sciences Daffodil International University Dhaka Bangladesh; ^2^ Department of Clinical Pharmacy, College of Dentistry & Pharmacy Buraydah Private Colleges Buraydah Saudi Arabia; ^3^ Department of Pharmacognosy, College of Pharmacy Prince Sattam Bin Abdulaziz University Al‐Kharj Saudi Arabia; ^4^ Department of Pharmacognosy, Faculty of Pharmacy Egyptian Russian University Cairo Egypt; ^5^ Department of Pharmaceutics KVSR Siddhartha College of Pharmaceutical Sciences Vijayawada Andhra Pradesh India; ^6^ Institute of Pharmaceutical Research GLA University Mathura Uttar Pradesh India; ^7^ Department of Pharmaceutical Chemistry Anurag Pharmacy College Suryapet Telangana India; ^8^ Department of Pharmaceutics, School of Pharmacy Anurag University Hyderabad Telangana India; ^9^ Department of Pharmacy Practice, Sri Ramachandra Faculty of Pharmacy Sri Ramachandra Institute of Higher Education and Research (DU) Chennai Tamil Nadu India; ^10^ Department of Pharmaceutical Analysis, MB School of Pharmaceutical Sciences Mohan Babu University (Erstwhile Sree Vidyaniketan College of Pharmacy) Tirupati India; ^11^ Department of Pharmaceutics, School of Pharmaceutical Sciences Vels Institute of Science, Technology and Advanced Studies (VISTAS) Chennai Tamil Nadu India; ^12^ Department of Pharmaceutics, School of Pharmacy Sathyabama Institute of Science and Technology Chennai Tamil Nadu India; ^13^ Department of Clinical Laboratory Sciences, College of Applied Medical Science King Khalid University Abha Saudi Arabia

**Keywords:** cancer, ferroptosis, lipid peroxidation, molecular signalling pathways, natural agents

## Abstract

Ferroptosis, a controlled cell death influenced by iron‐dependent lipid peroxidation, presents potential therapeutic targets for cancer treatment due to its unique molecular pathways and potential drug resistance. Natural compounds, such as polyphenols, flavonoids, terpenoids and alkaloids, can influence ferroptosis via important signalling pathways, such as Nrf2/Keap1, p53, and GPX4. These are promising for combinational therapy due to their ability to cause ferroptotic death in cancer cells, exhibit tumour‐specific selectivity and reduce systemic toxicity. Furthermore, these compounds, when combined with traditional chemotherapy or radiation therapy, can enhance therapeutic efficacy and overcome resistance. Natural compounds targeting ferroptosis offer innovative cancer treatment, particularly for resistant malignancies, due to their ability to interact with signalling pathways and produce specific cytotoxic effects. This review explores natural compounds' molecular mechanisms controlling ferroptosis in cancer, their interactions with traditional chemotherapeutics, translational hurdles, and clinical application directions, potentially leading to novel nature‐inspired anticancer treatments. Further research and clinical trials are needed to confirm the safety, bioavailability, and effectiveness of ferroptosis medicines, focusing on improved formulation and transport methods.

## 1 Introduction

Ferroptosis is a novel type of cell death that significantly differs from conventional processes like necrosis, autophagy and apoptosis [1]. Ferroptosis is characterised by intracellular iron deposition, which results in oxidative stress (OS), the production of reactive oxygen species (ROS) and the accumulation of lipid peroxide (Lipid‐OOH) [2]. The main cause of this issue is the decrease in glutathione (GSH) production and glutathione peroxidase 4 (GPX4) activity [3]. High iron ion concentrations lead to the accumulation of harmful lipid ROS, causing cell death [4]. Erastin can cause an iron‐dependent, non‐apoptotic death pattern in human BJ fibroblasts [5]. Erastin regulates System Xc^−^ activity, preventing GSH production and disrupting redox equilibrium, leading to L‐ROS deposition. Ras‐selective lethal 3 and RSL5 selectively kill human BJ fibroblasts without apoptosis [6]. A study utilised ferrostatin‐1, the first ferroptosis inhibitor, to explain the iron‐dependent, non‐apoptotic cell death induced by erastin and RSLs [1]. Ferroptosis, an endogenous anticancer mechanism mediated by the tumour suppressor gene p53, offers a new perspective on multidrug resistance in various malignancies [7, 8]. GSH, an antioxidant, can enhance tumour resistance in chemotherapy medications, while System Xc^−^ inhibitors, such as sulfasalazine, reverse drug resistance by increasing drug transport and blocking GSH formation [9].

Natural compounds provide more regulatory targets, a stable structure, less toxicity, less expense, and greater accessibility than traditional ferroptosis inducers [10]. The ferroptotic process is primarily attributed to the significant accumulation of lipid peroxidation products, which are primarily influenced by iron. Ferroptotic cell death can be prevented by iron chelators and small‐molecule lipophilic antioxidants. System Xc^−^ and GPX4 are crucial regulators [3, 11]. Tumour cells require high reactive oxygen levels and hypermetabolism for function, but they also have a higher risk of ferroptosis compared to healthy cells. Ferroptosis is a process that forms a natural barrier for the body, activates tumour suppressors, and effectively prevents the spread of cancer [7, 12]. Ferroptosis targeting may provide novel therapeutic options for patients who are resistant to traditional treatments or are indifferent to them. Furthermore, it is crucial for immunity and tumour suppression [13, 14]. Ferroptosis counters apoptosis's inability to generate a sufficient immune response by producing proinflammatory factors, which promote tumour immune response and inhibit tumour growth [15]. Furthermore, natural compounds may be effective in controlling ferroptosis and potentially treating related diseases. Bioactive peptides are substances that are considered antioxidants. The functional amino acid sequences of these peptides activate them to react with any oxidant, preventing or reducing its harmful effects [16]. In our review, we discussed the role of the therapeutic targeting of ferroptosis modulation by natural agents in cancer through a molecular signalling pathway.

## 2 Ferroptosis and Cancer Suppression

**FIGURE 1:**
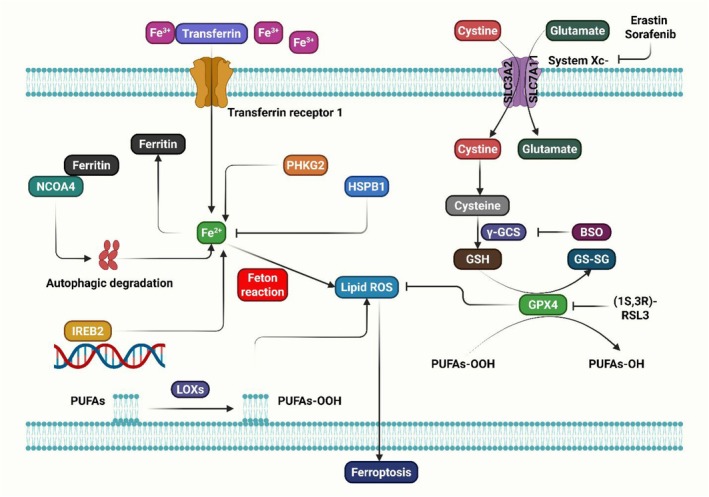
Ferroptosis, a cell death mechanism, occurs when system Xc^−^ or GPX4 activity is inhibited, potentially due to lipid ROS, PUFAs peroxidation, or excess iron. GSH, glutathione; NCOA4, nuclear receptor coactivator 4; PUFAs, polyunsaturated fatty acids; ROS, reactive oxygen species.

Tumour formation involves controlled cell death processes such as apoptosis, autophagy, and necrosis, among others [17, 18, 19, 20]. Ferroptosis inhibits carcinogenesis, with siRNA knockdown of GPX4 reducing protein levels, leading to renal cell carcinoma death and lipid ROS production [3]. Vitamin E and deferoxamine can prevent ferroptosis, while blocking SLC7A11, a cystine/glutamate antiporter, can trigger the process [1]. High levels of SLC7A11 expression are found in human malignancies, which prevent cancer cells from undergoing ferroptosis [21, 22]. The suppression of SLC7A11, both pharmacologically and genetically, leads to ferroptotic cell death and increases cisplatin cytotoxicity in cisplatin‐resistant HNC cells [23]. Jiang et al. revealed that p53 partially restricts tumour development by inhibiting SLC7A11 expression, leading to ferroptosis, cell cycle arrest, senescence, and apoptosis [7]. In response to ROS‐induced stress, the p533KR mutant, an acetylation‐defective variant, may control SLC7A11 expression and promote ferroptosis without resulting in apoptosis, senescence or cell cycle arrest [7]. The early‐onset spontaneous thymic lymphoma development in p53‐null mice did not impact p53 3KR animals [24]. Pro‐B cell lymphomas consistently failed to kill p533KR/3KRXRCC4−/−mice, unlike p53−/‐XRCC4−/−mice [25]. Ferroptosis activation may aid in treating various human cancer types [5]. Erastin, when used at low cytotoxic dosages, significantly enhances the anticancer effects of doxorubicin and cytarabine, two primary chemotherapy medications, in HL60 cells [26]. Because it can cause ferroptosis by blocking system Xc^−^ activity, the multikinase inhibitor sorafenib has been authorised to treat renal cell cancer and other diseases [11]. HCC patients develop sorafenib resistance, resulting in a poor prognosis [27, 28]. Research shows that various compounds, including RSL3, RSL5, ART, DHA, and small‐molecule inducers, can cause ferroptotic cell death (Figure 1) in cancer cells [29, 30, 31, 32]. Ferroptosis is a main factor in tumour suppression, cancer development, and therapy response. Ferroptosis, a rare tumour induced by erastin or RSL‐3, can be identified [3].

## 3 Signalling Pathway of Ferroptosis

### 3.1 Iron Metabolism

**FIGURE 2:**
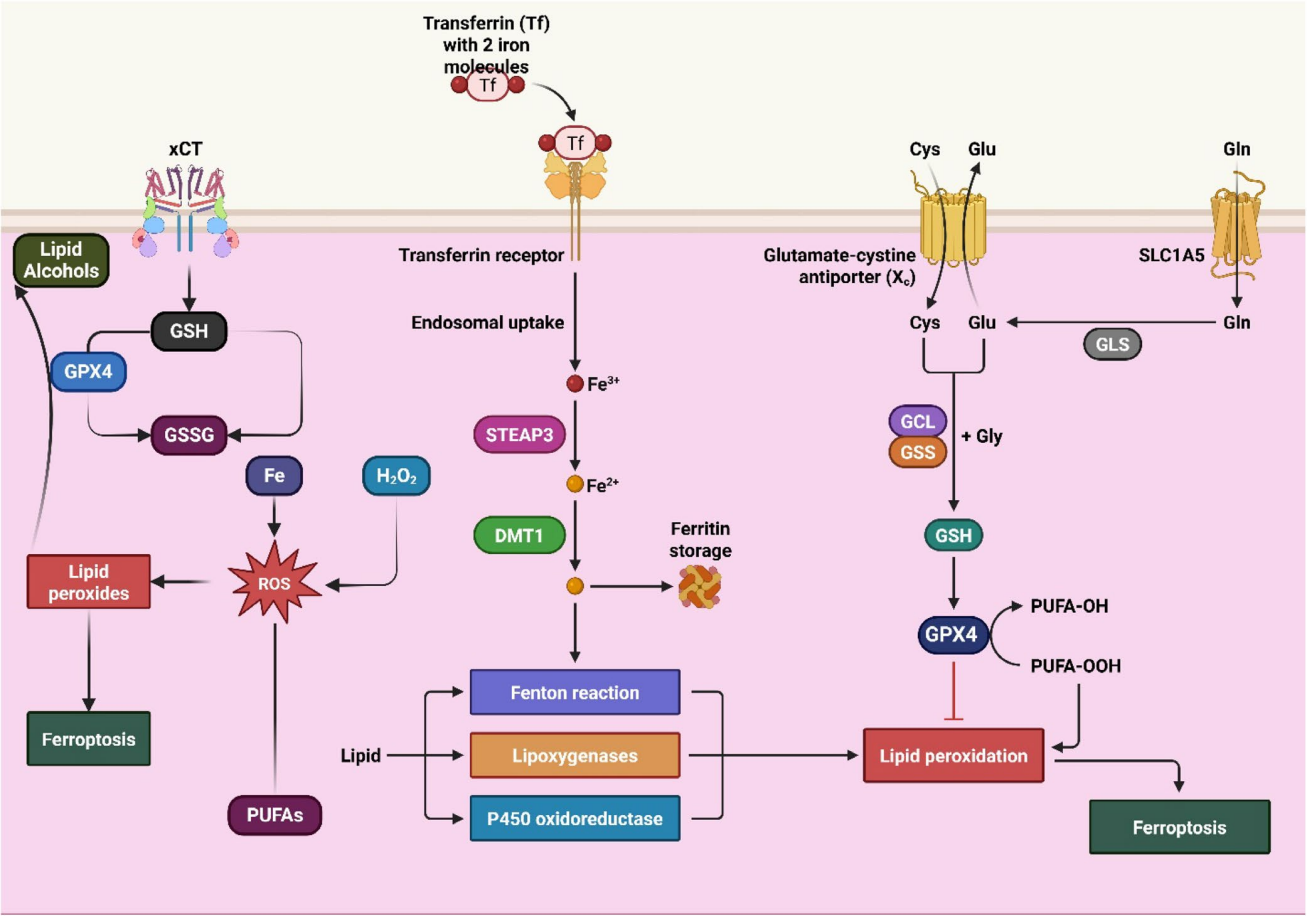
Iron metabolism regulates ferroptosis through key signalling pathways.

Ferrous ions activate iron‐containing enzymes in ferroptosis (Figure 2), generating lipid peroxides and providing energy to cells through the Fenton reaction, while iron chelators can significantly inhibit ferroptosis [33]. Iron metabolism involves absorption, export, storage, and use, controlling intracellular iron levels through dietary absorption and macrophage recycling from senescent erythrocytes, regulated by serum transferrin [33]. STEAP3, a prostate antigen, converts Fe^3+^ to Fe^2+^ in endosomes, then passes through SLC11A2/DMT1 [34]. Cancer cells typically use the NFS1‐ISCU enzyme to use iron, with excess Fe^2+^ in mitochondria causing ferroptosis, enzyme inactivation, and poor iron metabolism [35, 36, 37]. PCBP stores excess Fe^2+^ in cells via SLC40A1 (FPN), then transfers it to ferritin (FTH1/FTL), which is then exported by exosomes via PROM2 [38]. Iron metabolism stages may be targeted by medications to combat cancers, with ferroptosis, caused by iron overload, preventing tumour cell growth and multiplication [39, 40]. Ferroptosis is a process that occurs in head and neck cancers, which is primarily mediated by the silencing of PCBP1 [41]. Eratin, through IREB2, regulates ferrous ion levels, leading to ferroptosis in BC and fibrosarcoma [1, 42]. FTH is destroyed by NCOA4‐mediated ferritinophagy, which attracts it to lysosomes [43]. Lysosomal activity and NCOA4 activation increase ferrous ion levels, thereby promoting ferroptosis [44, 45]. Iron storage proteins, such as ferritin, play an essential role in preventing ferroptosis, as they enhance ferritinophagy and inhibit cytosolic glutamate oxaloacetate transaminase 1 in neuroblastoma cells [46, 47]. Nrf2 regulates iron metabolism through HO‐1, potentially causing non‐canonical ferroptosis if overactivated. However, modest HO‐1 overexpression may enhance its antioxidant properties (Table 1) [48, 58].

**TABLE 1:** Natural products with ferroptosis‐inducing potential and their therapeutic implications in cancer treatment.

Natural agents	Type of cancer	Mode of action	Finding	Ref.
Withaferin A	Neuroblastoma	Inactivated glutathione peroxidase 4 and enhanced Fe^2+^ by triggering Keap1 to activate HO‐1.	The nano‐targeted activation of dual ferroptotic pathways eliminated high‐risk neuroblastoma.	[48]
Solasonine	Hepatocellular carcinoma	Inhibition of GPX4 and GSS to enhance lipid ROS.	Increased ferroptosis of hepatoma carcinoma cells.	[49]
Ginkgetin	Non‐small cell lung cancer	Reduced SLC7A11 and GPX4 expression, Nrf2/HO‐1 inactivation	Increased effect of cisplatin in EGFR wild‐type NSCLC.	[50]
Erianin	Lung cancer	The activation of CaM led to an enhancement in Fe^2+^ levels after downregulating GPX4.	Suppressed LC cell growth and migration.	[51]
β‐Elemene	Colorectal cancer	Increased the expression of HO‐1 and TF to boost Fe^2+^ levels.	Induced ferroptosis and prevented EMT, making it sensitive to KRAS mutant CRC cells.	[52]
Cucurbitacin B	Nasopharyngeal cancer	Reduced GPX4 expression and collected iron ions.	Induced ferroptosis in human nasopharyngeal carcinoma cells	[53]
Oleanolic acid	Cervical cancer	Increased iron ions and ACSL4 expression lead to the accumulation of ROS and MDA.	Suppressed cervical cancer HeLa cell proliferation.	[54]
6‐Gingerol	Lung cancer	Reduced the expression of GPX and stimulated ferritinophagy by upregulating LC3‐II and NCOA4.	Inhibited Ubiquitin‐Specific Peptidase 14, thereby promoting ferroptosis.	[55]
Gambogic acid	Melanoma	Decreased SOD activity via upregulating p53 to block SLC7A11 and GPX4.	Induced ferroptosis in melanoma cells during the EMT.	[56]
Oridonin	Oesophageal cancer	Inhibited GSH formation and decreased GPX4 activity, and reduced GGT1 activity.	Induced ferroptosis by suppressing the gamma‐glutamyl cycle in TE1 cells.	[57]

### 3.2 Lipid Metabolism

Non‐enzymatic lipid peroxidation involves intracellular radicals, generating hydroxyl radicals and forming lipid peroxy radicals (LOO–) after removing hydrogen from PUFA at the phospholipid sn2 site. LOO, when interacting with PUFA or Fe^2+^, can produce lipid peroxide (LOOH) and a new lipid radical, initiating a chain reaction. Artemisinin increases ferroptosis risk in cancer cells by increasing cellular free iron and lipid peroxidation, causing increased membrane permeability and decreased integrity [59]. ACSL4 is considered crucial for ferroptosis due to its role in the peroxidation of PLs, which is more important than FAs [60, 61]. Arsenic trioxide is a substance that targets ACSL4 and can lead to the development of anticancer ferroptosis [62]. Popular ALOX inhibitors have RTA action, raising questions about the crucial role of ALOX in ferroptosis [63]. Ferroptosis is not caused by non‐enzymatic lipid peroxidation but by the enzymatic lipid peroxidation response [63, 64].

### 3.3 Oxidative Stress Pathway

Ferroptosis defence mechanism involves scavenging ROS and lipid peroxidation, regulated by pathways like Hsp90 and Hsp70, enhancing the regulatory network [7, 65, 66, 67]. Ferroptosis and lipid peroxidation can be prevented by systems like FSP1, CoQ10, BH4, and ESCRT‐III membrane repair, which do not rely on GPX4 [68, 69]. GPX4 and DHODH can enhance mitochondrial lipid peroxidation inhibition, unlike FSP1 and cytoplasmic GPX4 and FSP1, as DHODH primarily occurs in mitochondria [70, 71]. GTP cyclohydrolase 1 (GCH1), a key antioxidant, prevents ferroptosis by producing BH4 [72, 73, 74].

## 4 Effect of Natural Products in Ferroptosis

### 4.1 Artemisinin

Artemisinin has anticancer and antimalarial properties by upregulating NCOA4 and DMT1 levels, raising ferrous ion levels, and causing ferroptosis by downregulating GSH and GPX4 levels [30, 59, 75]. Artemisinin and its derivatives regulate 20 iron metabolism genes, thereby causing the formation of ROS [76]. Artesunate, when combined with sorafenib, can enhance the susceptibility of hepatocellular carcinoma cells to cisplatin resistance through ferroptosis inhibition [77]. Guo et al. investigated the potential anticancer properties of artemisinin, specifically ferroptosis, by controlling iron metabolism, producing ROS, and triggering ER‐stress. The primary antineoplastic mechanisms of artemisinin are ferroptosis, DNA damage, tumour angiogenesis suppression and cell cycle inhibition [78]. Artemisinin induces ferroptosis in cancer cells. It slows cancer progression and has potent anticancer actions [79]. Additionally, artemisinin has gained recognition as a potential anticancer agent. ART1, an analog of artemisinin, can cause ferroptosis in cancer cell lines. SAR research reveals that ART1 targets the HSD17B4 protein, an enzyme in the breakdown of fatty acids, without interfering with its function [80]. Artemisinin combats cancer and malaria. Its derivatives are effective anticancer medications, preventing cancer cell invasion, promoting cell cycle arrest, and increasing apoptosis. Artemisinin‐induced ferroptosis, a novel cell death mechanism, is triggered by ferritin content [81]. Moreover, artemisinin derivatives DHA and ART show anticancer effects by promoting ROS‐dependent apoptosis and ferroptosis in A549 cells, while NAC and ferrostatin‐1 partially reverse these effects [82]. Another study revealed that 30 iron‐related genes significantly influence artemisinins log_10_IC_50_ values, suggesting ferroptosis as a cell death pathway, making ferroptosis‐inducing drugs attractive for cancer therapy [76].

### 4.2 Kaempferol

Kaempferol (KP) is being developed as a potential preventative agent for hepatocellular carcinoma (HCC). It interacts with the body's pleiotropic proteins and has been shown to decrease OS, thereby reducing the risk of HCC [83]. Yuan et al. demonstrated the potential role of ferroptosis in neuronal damage caused by oxygen–glucose deprivation/reperfusion, utilising KP. Results showed that OGD/R reduced antioxidant levels, increased lipid peroxidation and caused ferroptosis. KP increased antioxidant capacity, inhibited lipid peroxidation and activated Nrf2/SLC7A11/GPX4 signalling. However, ML385 reduced Nrf2, preventing KP's protective effects [84]. KP exhibits antioxidant and anti‐inflammatory properties, protecting against OS, activating AMPK, promoting autophagy, and inhibiting ferroptosis in HepG2 cells. Oral KP treatment in mice also showed protective benefits against hepatotoxicity caused by CCl4 therapy [85]. Acetaminophen‐induced liver damage (AILI) is a public health concern linked to ferroptosis, a lipid peroxide‐induced cell death. KP has anti‐inflammatory and antioxidant properties. It improved hepatic iron overload and OS in mice, reduced hepatic damage in AILI animals, and protected L02 cells from APAP‐induced hepatotoxicity [86]. KP protects HUVECs from ferroptosis caused by RSL3. It decreased ROS accumulation and exhibited ferroptotic morphological alterations [87].

### 4.3 Baicalein

Baicalein is a compound that is used as a cancer treatment for CRC. It inhibits ferroptosis production in human CRC lines HCT116 and DLD1, reducing cell viability and GPX4 expression, supporting its therapeutic use against CRC [88]. Additionally, baicalein inhibited chondrocyte ferroptosis, reducing pain sensitivity and promoting AMPK/Nrf2/HO‐1 signalling activity. It may be a useful treatment approach for OA, as it reduces the development of OA by preventing chondrocyte ferroptosis [89]. Baicalein inhibits erastin‐induced ferroptosis in pancreatic cancer cells by preventing lipid peroxidation, glutathione depletion, and ferrous iron synthesis, suggesting potential treatment for ferroptosis‐induced tissue damage [90]. A study found baicalein's impact on melanocyte ferroptosis in vitiligo. It reduced RSL3 toxicity, reducing cell death, mitochondrial malfunction, ROS generation, and iron ion buildup. In RSL3+FAC treatment, baicalein increased GPX4 and decreased TFR1, protecting melanocytes against ferroptosis [91]. Baicalein induces ferroptosis in CRC cells by inhibiting the Janus kinase 2/STAT3 signalling pathway and reducing GPX4 expression, ultimately causing ferroptosis in these cells [88]. Additionally, baicalin has anticancer properties against bladder cancer by causing cell death and ROS accumulation. Baicalin's anticancer effects were mediated by ferritin heavy chain 1, causing ferroptosis. Overexpression of FTH1 negated baicalin's anticancer benefits, suggesting a potential bladder cancer therapy [92]. Baicalin enhanced cisplatin‐induced HK2 cell injury and reduced renal dysfunction. It inhibited ferroptosis and phospholipid peroxidation in AKI by lowering the expression of ALOX12. The regulatory impact of baicalein was comparable to that of ALOX12 silencing, and baicalein is a medication that effectively treats AKI [93]. Furthermore, Wen et al. demonstrated the antitumour properties of baicalin on osteosarcomas, revealing that baicalin inhibits tumour cell proliferation, induces ferroptosis, and produces MDA. Ferrostatin‐1, a ferroptosis inhibitor, counteracts baicalin's anti‐OS effects by interacting with Nrf2, a key regulator of ferroptosis, thereby promoting ubiquitin degradation and xCT production. This research shows baicalin's anti‐OS action via a new Nrf2/xCT/GPX4‐dependent regulation axis, making it a viable option for treating OS [8]. Baicalin's anticancer effects are mediated by ferritin heavy chain 1, potentially leading to bladder cancer treatment by causing FTH1‐dependent ferroptosis [92]. Additionally, baicalin upregulates the p53 gene, disrupting iron homeostasis and antioxidant defence, leading to iron accumulation, lipid peroxide aggregation, and ferroptosis activation in gastric cancer cells. It increases susceptibility to oxaliplatin treatment by activating ferroptosis through various routes [94]. Moreover, baicalin suppresses various malignancies and increases their treatment susceptibility. The combination of 5‐Fu and baicalin also increased ferroptosis in gastric cancer, slowing the progression and increasing 5‐Fu [95]. Furthermore, baicalin reduced the viability of SGC‐7901 cells, but this reduction was lessened by the Fer‐1 intervention. It increased GSH activity and reduced ROS levels in SLC7A11‐overexpressing cells. It also inhibits the growth of SGC‐7901 cells through p53/SLC7A11‐mediated ferroptosis [96].

### 4.4 Luteolin

Luteolin induces ferroptosis in prostate cancer cells by activating TFEB, affecting cell death, autophagy, and TFEB nuclear translocation, while enhancing ferritinophagy [97]. Additionally, luteolin and erastin effectively reduced colon cancer cell viability and growth, increasing ferroptosis and decreasing glutathione peroxidase 4 (GPX4) expression. HIC1 overexpression promoted ferroptotic cell death and enhanced GPX4 inhibition [98]. A new antioxidant flavonoid complex called Lu‐Mn nanozyme, formed by chelating luteolin with manganese ions, has shown potential in cancer treatment. In vitro tests showed that it efficiently catalyses hydroxyl radical production, causing tumour cell apoptosis and ferroptosis. Lu‐Mn has a good safety record and strong antitumour effectiveness [99]. Luteolin reduces liver cell damage caused by sorafenib, a protein kinase inhibitor. It increased glutathione expression, chelated iron, and inhibited mitochondrial membrane potential. It also activated the Nrf2‐associated pathway, reducing sorafenib‐induced ferroptosis. It could be used as an adjuvant to sorafenib to reduce liver damage in individuals with hepatocellular carcinoma [100]. Moreover, luteolin, a compound with strong anti‐inflammatory and cellular ferroptosis‐regulating properties, was found to contribute to inflammation and ferroptosis during CAG. A study using a CAG rat model demonstrated that luteolin decreased inflammation by blocking the AGE‐RAGE signalling cascade and inhibited CAG by downregulating ACSL4 and NOX1 expression levels [101].

### 4.5 Apigenin

Apigenin protects against various cancers, but it stimulates ferroptosis and autophagy in Ishikawa cells. It may promote autophagy by downregulating AMPK and upregulating Beclin 1 and limit tumour development by ferroptosis in vivo [102]. Apigenin has antioxidant properties and can reduce or prevent damage from toxins. Exposure to DEHP causes ferroptosis by raising ROS, interfering with lipid peroxidation, and iron homeostasis. Apigenin supplementation reduces these alterations, inhibiting intracellular iron buildup and activating GPX4 [103]. A study on non‐obese diabetic (NOD)/LtJ female mice found that apigenin, a phytoestrogen, reduces symptoms and development of Sjögren's syndrome by preventing ferroptosis in salivary gland epithelial cells. It inhibited ferroptosis and immune infiltration, increased salivary production, and decreased OS gene ATF3 expression. It also counteracted the effects of IFN‐γ treatment, which increased ATF3 expression and ferroptosis while downregulating AQP5, SLC7A11 and GPX4 expression [104]. Apigenin can prevent ferroptosis in mice fed high‐fat diets. It can restore lysosomal membrane permeability, reducing ROS generation and preventing iron from leaking into the cytoplasm. It also prevents ferroptosis caused by high‐fat diets and palmitic acid [105]. Mitochondria supply adenosine triphosphate for tumour cell growth. Inducing mitochondrial damage can reduce tumour cell bioactivities, increase drug sensitivity and reverse drug resistance. Synthesised lipid nanoparticles called Lip@AF, co‐delivered with FdUMP and apigenin, can disrupt the balance of ROS and glutathione. αCD276‐Lip@AF, an immune checkpoint inhibition reagent, was modified to inhibit tumour growth in vivo, providing new paths for improving chemotherapy sensitivity and high‐performance chemoimmunotherapy [106]. Metal‐polyphenol nanostructures, like nFeAPG, have been formed using a coordinated strategy involving Fe^3+^ and apigenin. In vitro studies showed nFeAPG can cause cell death by increasing OS and suppressing the antioxidative system. It also improves the immunological environment [107]. Apigenin has anticancer effects in various cancer types. It induces senescence, anoikis and necroptosis by downregulating drug‐resistance mediators and stimulating ROS production [108].

### 4.6 Epigallocatechin Gallate

Green tea's medicinal benefits are primarily due to its main polyphenolic component, epigallocatechin gallate (EGCG). Polyphenolic substances exhibit diverse biological and pharmacological properties, including immunomodulatory, chemoprotective, and anticancer activities [109]. A study investigates the potential therapeutic applications of EGCG in treating ferroptosis. It inhibited cell growth, increased ACSL4 levels, and decreased GPX4 and SLC7A11 expression. It also downregulated tsRNA‐13,502 and altered the expression of ferroptosis regulators, leading to ferroptosis in NSCLC cells [110]. EGCG induces ferroptosis by promoting the breakdown of GPX4 protein and altering iron metabolism [111]. Additionally, EGCG decreased STAT1 expression in A549 cells, a key oncogene in lung cancer. It inhibited ferroptosis, a non‐apoptotic cell death mediated by STAT1, and inhibited lung cancer cell proliferation. Leptin increased the gut microbiota and STAT‐SLC7A11 pathway, enhancing lung carcinogenesis [112]. High concentrations of EGCG, which regulates mitochondrial glutamine metabolism, inhibited glutathione peroxidase, leading to tumour cell ferroptosis [113].

### 4.7 Gallic Acid

**FIGURE 3:**
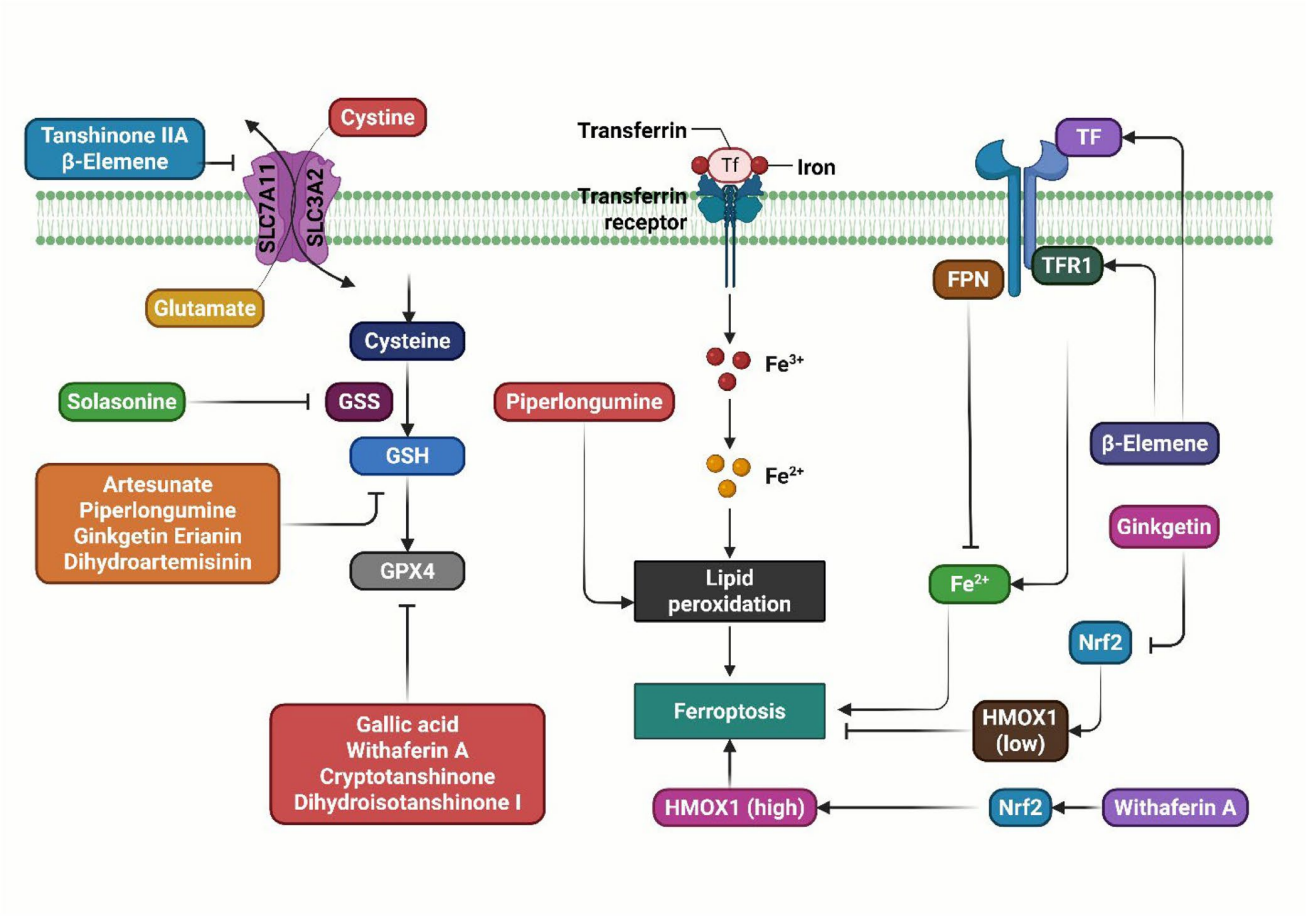
Illustration of the role of natural products in inducing ferroptosis. GPX4, glutathione peroxidase 4; GSH, glutathione; GSS, glutathione synthetase; Nrf2, nuclear factor erythroid 2‐2‐related factor 2.

Gallic acid (GA) is found in fruits, vegetables, mushrooms, and herbs [114]. Polyphenols with anticancer effects include GA and its derivatives [115]. Khorsandi et al. found that GA treatment reduced the cell death rate and GPX4 activity (Figure 3), while concurrent treatment increased ROS generation and lipid peroxidation. The study suggests a novel cancer treatment method that combines ferroptosis with radiation therapy [116]. Khorsandi et al. revealed that GA and low‐level laser generated more ROS than GA alone. The findings suggest that low‐level laser irradiation is insufficient to destroy both normal and cancerous human cells [116]. GA, a novel HC ferroptosis inducer, inactivates the Wnt/β‐catenin pathway in HepG2 cells, limiting the production of ferroptosis‐related proteins SLC7A11 and GPX4, potentially promising for the clinical management of HCC [117]. Additionally, GA is linked to iron‐dependent cell death processes, with deferoxamine, an iron chelator, potentially reducing GA‐induced cell death. Necrosulfonamide, an inhibitor of MLKL, increases cancer cell sensitivity to GA, enhancing its effectiveness [118]. Chen et al. demonstrated the mechanisms causing ferroptosis‐related radiosensitisation of SL‐GAC in NSCLC cells. SL‐GAC was formed to increase GA bioavailability. The combination inhibited NSCLC cell proliferation, migration, OS, and mitochondrial dysfunction. However, it also caused ferroptosis, which was reversed by ferrostatin‐1 [119].

### 4.8 Isothiocyanates

**FIGURE 4:**
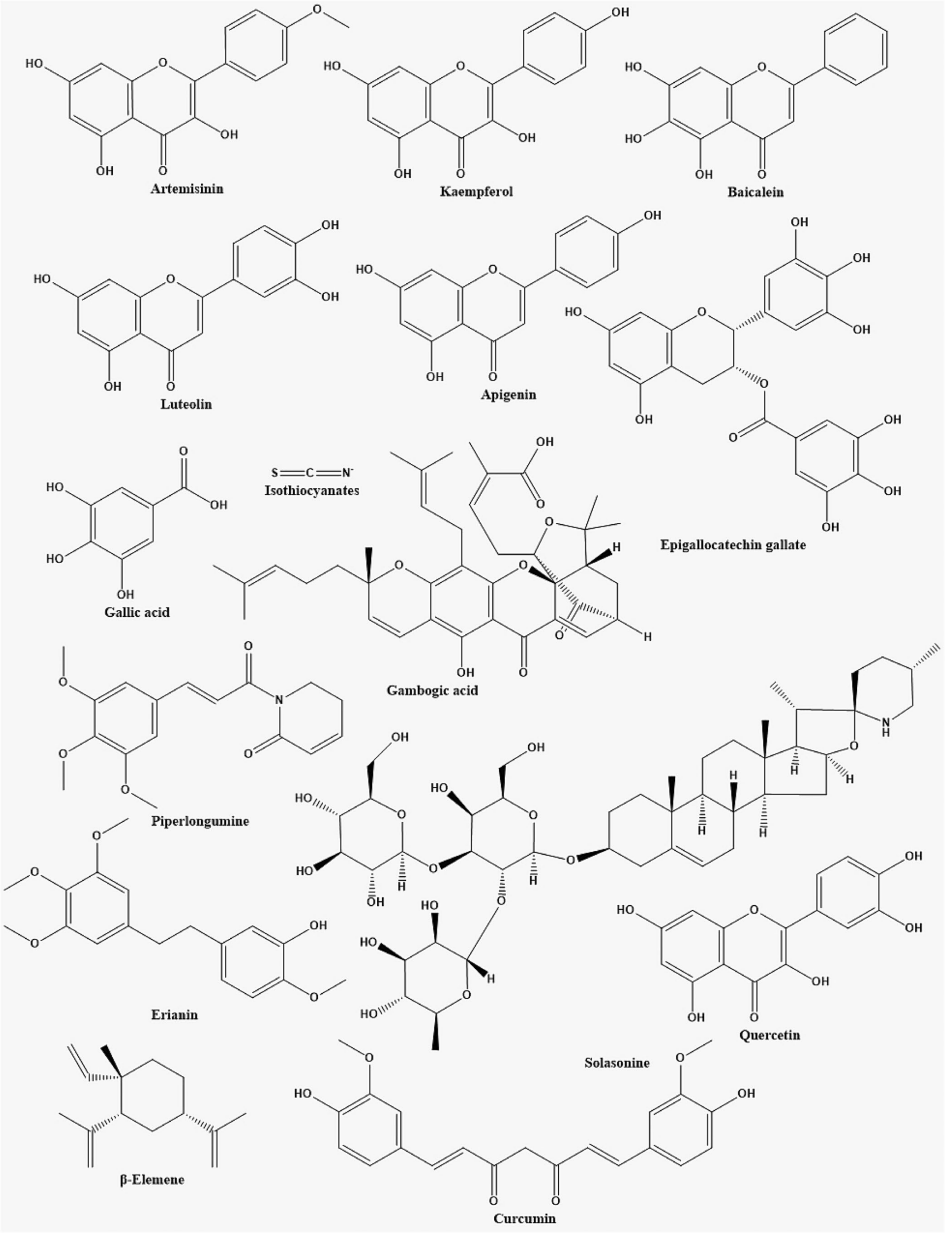
Chemical structures of natural compounds that modulate ferroptosis and exhibit therapeutic potential in cancer treatment.

Isothiocyanates (Figure 4), organic sulfur compounds, have anticancer properties [120]. A study indicates that PEITC and cotylenin A can induce pancreatic cancer cells to produce ROS and die. N‐acetylcysteine, DFO, and ferrostatin‐1 prevent cell death, while necrosis and apoptosis inhibitors do not work effectively [121]. PEITC causes ferroptosis in human osteosarcoma by activating the MAK signalling pathway, altering iron metabolism, and disrupting redox homeostasis. Isothiocyanates can control iron metabolism and intracellular redox balance, causing ferroptosis in tumour cells and apoptosis, potentially offering a way to manipulate ferroptosis in tumour therapy [122]. Moreover, a study utilised a hybrid AR antagonist and a GSH synthesis inhibitor, buthionine sulfoximine, to inhibit androgen receptor activation and promote ferroptosis in CRPC cells. This combination improves anti‐PCa properties, reduces lipid peroxidation and restores cell viability [123]. Phenylethyl isothiocyanate and sulforaphane exhibit pleiotropic anti‐PCa properties. The combination reduced cell viability in multiple PCa cell lines, while non‐cancerous prostatic RWPE‐1 cells were less affected. The findings suggest that 2‐63 and BSO work together by improving drug accessibility, increasing iron availability and possibly influencing the activity of glutathione peroxidase 4 [124].

### 4.9 Gambogic Acid

Researchers are investigating the potent properties of gambogic acid, an anticancer ingredient derived from a traditional Chinese medication [121]. Pan et al. [125] discovered that gambogic acid disrupts cellular redox equilibrium, leading to ferroptosis and apoptosis due to increased ROS production by inhibiting the thioredoxin system. Researchers found that gambogic acid enhanced the antitumor activity of cancer medications like docetaxel and enzalutamide, suggesting that combining gambogic acid with existing treatments could improve tumour therapeutic benefits [126, 127]. Furthermore, Lan et al. investigated the effects of gambogic acid on HepG2 and MCF‐7 cells in vitro. They found that gambogic acid has a strong tumour‐killing ability, but ferroptosis inhibitors counteracted this effect. Gambogic acid's acid‐induced ferroptosis indicators included ROS and mitochondrial membrane potential changes. Ferrostatin‐1 and lipostatin‐1 prevented gambogic acid‐induced MDA accumulation. The study highlights the importance of the Nrf2‐mediated GSH/Trx dual antioxidant system in ferroptosis [128].

### 4.10 Piperlongumine

Piperlongumine (Figure 4), derived from the plant's dry root, is known for its pharmacological benefits, including analgesia, anxiety reduction, anticancer, antiplatelet agglutination, antifungal, and antidepressant properties [129]. Additionally, piperlongumine, when combined with SSZ and CN‐A, effectively destroys human pancreatic cancer cells by increasing ROS levels, reducing ferroptosis [130]. Piperlongumine may enhance ROS generation and cell death in cancer cell lines through Nrf2‐mediated mechanisms, as it interacts with Keap1 and increases heme oxygenase 1 production [131]. Furthermore, piperlongumine, when combined with HO‐1, can enhance ROS generation and cell death in cancer cells without causing harm to healthy cells. HO‐1 can increase ferroptosis caused by erastin by producing iron ions, but further experimental confirmation is needed to determine its role in piperlongumine ferroptosis. Piperlongumine causes ferroptosis by controlling iron metabolism and raising ROS levels [132]. A study evaluates the anticancer effects of piperlongumine and CB‐839 on oral squamous cell carcinoma (OSCC) cells. It can cause cell death, increase ROS and lipid peroxidation, and cause protein alterations. Fer‐1 and NAC can counteract piperlongumine's antiproliferation action, reducing ROS and LPO levels (Figure 3). Piperlongumine and CB‐839 can synergistically lower lipid peroxidation levels and cell viability, consuming significant glutathione. Piperlongumine and CB‐839 may provide a novel approach to treating OSCC due to their synergistic anticancer effect [133]. Another study found the impact of piperlongumine on glioblastoma multiforme. It assessed its effects on glioma cell survival and growth. It also suppressed glioma cell viability and proliferation, with the inhibitory effect restored by ferroptosis inhibitors. It suppresses glioblastoma multiforme growth by causing ferroptosis [134]. Piperlongumine inhibits cytosolic thioredoxin reductase (TXNRD1) and has been found to destroy cancer cells. It inhibits recombinant TXNRD1 irreversibly, with the UGA‐truncated variant resistant to it. This study suggests that blocking TXNRD1 could enhance cancer cell ferroptosis [135].

### 4.11 Erianin

Erianin, a naturally occurring substance from *Dendrobium chrysotoxum* Lindl., exhibits anticancer activities [136]. Additionally, erianin has potential anticancer properties, especially in lung cancer. It inhibits lung cancer cell proliferation by affecting cell cycle progression and apoptosis. It also interferes with pyrimidine metabolism, a key regulator of the pyrimidine process. Erianin's primary target is mTOR, which blocks downstream effectors like S6K and CAD. By reducing mTOR activation and interfering with pyrimidine metabolism, erianin may prevent lung cancer cell growth [137]. Erianin therapy effectively prevented cell death by blocking a specific signalling pathway, demonstrating its role in preventing ferroptosis [51]. Miao et al. found that erianin enhanced the thermal stability of human KRASG13D cells. In vitro results showed it inhibited migration, invasion, and EMT and caused ferroptosis. Autophagy inhibitors and ATG5 knockdown reduced erianin‐induced ferroptosis [138]. Erianin suppresses cancer cell activity. A study isolated CD44+/CD105+ HUCSCs and found that erianin caused OS injury, reduced proliferation, invasion, angiogenesis, tumourigenesis and increased mRNA N6‐methyladenosine modification in HUCSCs [139]. Furthermore, Zheng et al. investigated the potential of erianin to treat CRC by causing ferroptosis. It prevented carcinogenesis and CRC cell proliferation in an animal model. It also caused ferroptosis in CRC cells, causing a rise in lipid peroxides and Fe^2+^ levels. Ferroptosis inhibitors effectively reversed the cytotoxicity of erianin [140]. Iron increases tumour cell proliferation. Erianin is linked to OS in HCC. After 24 h of increasing erianin concentrations, survival rates declined. Combining ferroptosis inhibitors with erianin increased cell survival rates. Erianin inhibited the expression of SLC7A11 and GPX4 and activated the JAK2/STAT3 pathway, suppressing tumour growth without harm [141]. Erianin controls cyclin and apoptotic proteins, preventing OS cells from proliferating and migrating. In vivo, it can prevent tumour growth and induce OS ferroptosis. The study also uses cell assays to assess Erianin's impact on cell viability, proliferation, and migration [142].

### 4.12 Solasonine

Solasonine (Figure 4) can inhibit pancreatic cancer cell growth by affecting the AP‐2 alpha/Otubain 1/SLC7A11 axis [143]. A study investigated solasonine's antitumour mechanism in OSCC using lipidomics, cell, and animal models. It inhibited cell growth, reduced tumour volume, increased diglyceride and triglyceride levels and caused ROS production and mitochondrial damage [144]. Solasonine inhibits the expression of ACHE in NSCLC cells. A study used artificial intelligence to predict the target protein for solasonine and found that it had a negative regulatory effect on tumour cells. Solasonine blocks P38 MAPK [145]. Additionally, solasonine can cause ferroptosis in HCC cells by interfering with the GSH redox pathway through GPX4 [49]. Zeng et al. suggested that solasonine, a chemical linked to tumour suppression, may be a potential treatment for lung adenocarcinoma. It inhibits lung cancer cells, contributing to the accumulation of lipid peroxide, Fe^2+^, and ROS. It treats lung cancer due to its potential to inhibit ferroptosis [146].

### 4.13 Quercetin

A plant‐based flavonoid called quercetin (Figure 4) is found in large quantities in a variety of fruits and vegetables, including green leafy vegetables, apples, grapes, and onions. The compound effectively prevents the spread of multiple tumours and exhibits antiviral, anti‐inflammatory, antifibrotic, and antioxidant properties [147]. Quercetin promotes ferroptosis mediated by autophagy in GC cells. It significantly reduced tumour volume and cell viability, suppressing beclin1 and LC3B levels while lowering glutathione, malondialdehyde, and ROS. However, these effects were reversed by siATG5 [148]. Two studies suggest quercetin aids in the breakdown of lysosome‐dependent ferritin, releasing free iron and initiating ferroptosis [149, 150]. Wang et al. investigated the relationship between ferroptosis and lysosome function in quercetin's anticancer properties. The substance initiates lysosomal activation, enabling the release of free iron and ferritin, which are dependent on lysosomes [150]. Quercetin regulates ferroptosis and has antiproliferative effects on breast cancer cells. It increases iron, MDA, and carbonyl protein levels, leading to cell death. TFEB is poorly expressed in the cytoplasm and substantially expressed in the nucleus. Chloroquine and TFEB siRNA can prevent quercetin's pharmacodynamic effects. Quercetin induces iron death, resulting in the death of breast cancer cells [149]. Quercetin, a hormone found to alleviate depression in breast cancer patients, has been investigated for potential therapeutic targets. Zhu et al. on a BCRD mouse model demonstrated quercetin's ability to enhance lipid metabolism and inhibit genes associated with the disease. It increased proliferation in hippocampal neurons, decreased ferroptosis‐related indicators, and raised serotonin, dopamine, and noradrenaline levels. It also decreased CA153 and IL‐10 levels. However, overexpression of PTGS2 reversed these effects [151]. Quercetin may have potential anticancer activity in OSCC. It inhibited tumour growth by promoting ferroptosis via mTOR/S6KP70‐dependent mechanisms, suppressing SLC7A11 expression, causing lipid peroxidation, and decreasing GSH levels. Ferroptosis may represent a novel quercetin antitumour mechanism [152].

### 4.14 β‐Elemene

β‐elemene (Figure 4), produced from *Curcuma zedoaria* , induces apoptosis and reverses chemotherapy resistance in various malignancies. When treated with radiation, it reverses radioresistance in GC cells and decreases cell proliferation. It increases ROS, MDA, and Fe^2+^ levels, facilitating ferroptosis. β‐elemene targets the GPX4 pathway and induces ferroptosis, effectively controlling radioresistance in GC [153]. Chen et al. showed that CRC cells with KRAS mutations showed a good response to cetuximab and β‐elemene therapy. This combo therapy enhanced sensitivity by causing ferroptosis and preventing the epithelial‐mesenchymal transition [52]. The ferroptotic reaction, triggered by isoliquiritin in Glycyrrhiza uralensis, produces isoliquiritin. Research showed that isoliquiritin blocks NF‐κB signalling, modifies ferroptosis and can reduce doxorubicin resistance in breast cancer [154]. β‐elemene has antitumour effects on NSCLC. It leads to a rise in GPX4 lysosome degradation, enhancing ubiquitination and lysosomal localisation [155]. The researchers formed a polymer loaded with β‐elemene, which disrupts the tumour cell's antioxidant system and produces ROS, leading to ferroptosis. This system, combined with chemotherapy and iron death, could potentially inhibit breast cancer cells [156]. β‐elemene suppresses the growth of GC cells. In vitro, it reduced malignant cell activity in DDP‐resistant GC cells and enhanced drug sensitivity. Overexpressing ARF6, which was increased in these cells and tissues, may reverse the effects of β‐elemene therapy. In vivo, it may inhibit tumour growth through the exosomal METTL3‐m6A‐ARF6 axis, potentially reducing the growth of tumours resistant to DDP [157].

### 4.15 Curcumin

Curcumin prevents breast cancer cells from surviving. It increased intracellular Fe^2+^ levels, lipid ROS levels, and MDA accumulation, promoting ferroptosis in both cells [158]. Additionally, curcumin inhibits breast cancer and glioblastoma cells by controlling ferroptosis, which contributes to its ability to inhibit NSCLC. It promotes cell death while suppressing tumour development and division. Iron overload, GSH depletion and lipid peroxidation are distinctive ferroptotic alterations in curcumin‐induced antitumour effects. It also increases autolysosomes, raises Beclin1 and LC3 levels, decreases P62 levels, and causes mitochondrial membrane rupture and cristae decrease. Autophagy inhibitors siBeclin1 and chloroquine reduce the autophagy caused by curcumin and ferroptosis [159]. Curcumin reduces breast cancer cells' ability to proliferate, causing ferroptosis, an oxidative cell death. This is a target for chemotherapy in cancer treatment. Curcumin upregulates target genes like heme oxygenase‐1 (HO‐1), which facilitates ferroptosis. However, the inhibitor zinc protoporphyrin 9 can increase cell viability and decrease ferroptosis‐related phenomena. Curcumin causes ferroptosis in breast cancer cells [160]. Moreover, curcumin exhibits strong antitumour activity in GC, increasing cell death, decreasing viability, improving autophagy, and stimulating ferroptosis [161]. Follicular thyroid cancer (FTC) is aggressive and requires investigation into tumourigenesis mechanisms and treatment pathways. HO‐1, an enzyme controlling OS, is abnormally overexpressed in FTC, potentially activating the ferroptosis signalling pathway. Curcumin inhibits tumourigenesis by upregulating HO‐1 expression, inhibiting FTC cell growth and potentially influencing FTC carcinogenesis [162]. Firouzjaei et al. found 39 genes shared by ferroptosis‐related genes and 739 differentially expressed genes in CRC. The study identified 17 potential therapeutic targets and predicted medications targeting CRC‐DEGs. Curcumin therapy increased SLCA5 and CAV1 expression in human CRC cells, suggesting curcumin could control FRGs and potentially treat the disease [163]. Curcumin inhibited the viability of SW‐480 cancer cells, increased intracellular lipid peroxidation and suppressed JNK signalling. It may also have an anticancer effect on CRC by downregulating JNK signalling [164]. Furthermore, curcumin has been developed as a tumour‐targeting drug delivery system using mesoporous silica nanoparticles. The system, which has excellent water solubility and biocompatibility, effectively reduced gastric cancer in vitro and in vivo by inducing ferroptosis [165]. Curcumin can reverse sunitinib resistance in ccRCC. A model of ccRCC cells was formed, and the effects of sunitinib alone or curcumin plus sunitinib were confirmed using various assays. Curcumin prevented sunitinib‐resistant ccRCC cells from proliferating, suppressed ferroptosis‐related protein expression, increased ADAMTS18 gene expression, and decreased iron ion concentration. However, curcumin's inhibitory effect on sunitinib‐resistant cell lines was eliminated by ferroptosis inhibitors [166]. Ming et al. investigated the effects of curcumin on ferroptosis in CRC. The research used various tests, including clonogenic, wound healing, and tetrazolium bromide tests, to assess curcumin's effects on malignant CRC cells. Curcumin inhibited the growth of xenograft tumours, increased lactate dehydrogenase release and decreased lipid peroxide levels in CRC cells. It may bind p53, SLC7A11, and GPX4, indicating that curcumin may induce ferroptosis and have an inhibitory effect on CRC [167]. Curcumin's antitumour properties are limited by low bioavailability. The effectiveness of NL01, a drug that induces ferroptosis in ovarian cancer cells, is being enhanced. NL01 reduces ovarian cancer growth and causes iron mortality. Further research shows that ferrostatin‐1 partially reverses growth inhibition and enhances GPX4 expression downregulation [168]. Additionally, curcumin significantly inhibited CRC cell growth and reduced tumour volume in CRC mice. It increased the abundance of SCFA‐producing microorganisms, affecting the distribution of blood metabolites. A faecal microbiota transplantation experiment confirmed curcumin's effects, reducing tumour growth and increasing CD8+ T cell infiltration. Curcumin's anticancer properties were significantly diminished when antibiotics were used to reduce gut microbiota [169].

## 5 Applications of Natural Products for Ferroptosis

### 5.1 Ferroptosis and Natural Products in Immunotherapy

Tumour immunotherapy is a crucial treatment for malignancies as it stimulates the body's immune system, controlling the immune response and preventing tumour development [170, 171]. Tumours can form distinct immunosuppressive environments due to their immunogenicity and checkpoints [172, 173, 174, 175]. Despite Teffs binding to tumour cells, immunological checkpoints like PD‐L1 and CTLA4 aid tumour cells [176, 177, 178]. Tumour cells secrete substances that stimulate mast cells, which in turn inhibit the immune system and promote the growth of tumours [179, 180, 181]. Natural products have the potential to improve cancer immunotherapy effectiveness by altering the tumour's microenvironment. Ginsenoside Rg3, resveratrol, and capsaicin are substances that increase the body's exposure to DAMP, thereby increasing its impact on immunogenic cell death [182, 183, 184]. Researchers are exploring the link between ferroptosis and tumour immunity. Ferroptosis‐induced cancer cells can stimulate the tumour microenvironment and generate a positive feedback immunological response through the production of DAMPs [185, 186, 187]. Ferroptosis‐induced lipid peroxidation may enhance tumour immunotherapy by promoting dendritic cells to recognise tumour antigens [188].

### 5.2 Ferroptosis and Natural Products in Drug Resistance

Numerous studies have attempted to challenge drug resistance, a significant challenge in the treatment of malignancies. Recent research suggests that ferroptosis may be linked to medication resistance [189]. SLC7A11 is overexpressed in various cancers, with factors stabilising or upregulating it in cells resistant to sorafenib and cisplatin, potentially reversing tumour suppression [40, 189, 190, 191]. Drug‐resistant cells exhibit significantly higher levels of Nrf2, as it encodes numerous antioxidant system proteins [40, 58]. GPX4 suppression is a crucial strategy for addressing cancer's resistance to chemotherapy [192]. Ferroptosis targeting, induced by natural products, is a promising treatment for medication resistance, as demonstrated by curcumin analog in glioblastoma by downregulating GPX4 [193]. Artesunate, by blocking GPX4, can reverse sunitinib resistance and cause ferroptosis in renal cell cancer by causing the cancer to spread [194]. Natural substances like ungeremine, epunctanone, and soyauxinium chloride have been found to exhibit cytotoxicity towards drug‐resistant tumour cells through ferroptosis [195, 196, 197].

## 6 Conclusion and Future Perspectives

Ferroptosis presents a promising cancer treatment target, particularly for unresponsive cancers that traditional treatments fail to address. Natural substances like polyphenols, flavonoids, terpenoids, and alkaloids show promise in regulating ferroptosis through pathways like system Xc^−^, p53, Nrf2/Keap1, and GPX4. Ferroptosis targets cancer cells by modifying iron metabolism, lipid peroxidation and antioxidant defence mechanisms. Natural substances like polyphenols, flavonoids, terpenoids, and alkaloids offer insights into ferroptotic cell death. These substances are effective in combination therapy and standalone treatments due to their ability to work with current chemotherapeutics and exhibit selective cytotoxicity towards cancer cells. Despite the promising preclinical data, several difficulties need to be overcome before these findings can be effectively applied in clinical settings. Many natural compounds provide major challenges due to their unknown pharmacokinetic characteristics, low target selectivity, and poor bioavailability. Understanding the tumour microenvironment's impact on ferroptosis and potential off‐target effects is crucial for ensuring safety and effectiveness. Future research should focus on developing nanoformulations and drug delivery methods to enhance the bioavailability and tumour‐specific accumulation of natural ferroptosis inducers. To confirm the safety profiles and therapeutic advantages of these medicines, thorough in vivo research and well‐planned clinical trials are necessary. Integrating natural products into ferroptosis‐targeting strategies has the potential to revolutionise cancer treatment and improve patient outcomes. To fully utilise these discoveries in clinical practice, several obstacles must be overcome. Many natural agents have poor bioavailability and metabolic instability, which hinders their therapeutic efficacy. The review raises safety and selectivity concerns due to potential non‐specific cytotoxicity, off‐target effects, and disruption of redox equilibrium. The lack of clinical data or ongoing trials that directly link natural substances to ferroptosis highlights the need for additional validation, as the majority of the current evidence is based on preclinical investigations. Furthermore, the effectiveness and replicability of ferroptosis‐targeted treatments may be influenced by the heterogeneity of tumours and the complexity of the tumour microenvironment. Therefore, future studies should prioritise methods to overcome these limitations. Nanotechnology‐based delivery methods, structural modifications for enhanced pharmacokinetics and combination treatments with immunotherapeutic or conventional medicines can improve effectiveness and minimise side effects. The identification of predictive indicators of ferroptosis sensitivity is crucial for classifying patients who are most likely to benefit the most. Crucially, clinical trials are crucial to confirm the preclinical promise of natural medicines and determine their safety, ideal dosage and therapeutic potential in human tumours. Natural medicines modulating ferroptosis have potential for cancer treatment, but bioavailability, off‐target effects, and translational evidence must be addressed for full potential.

## Author Contributions

**Md. Al Amin:** conceptualization (equal), data curation (equal), resources (equal), validation (equal), visualization (equal), writing – original draft (equal), writing – review and editing (equal). **Mehrukh Zehravi:** conceptualization (equal), formal analysis (equal), investigation (equal), supervision (equal), validation (equal), visualization (equal), writing – original draft (equal), writing – review and editing (equal). **Sherouk Hussein Sweilam:** data curation (equal), resources (equal), validation (equal), visualization (equal), writing – original draft (equal), writing – review and editing (equal). **Patibandla Jahnavi:** data curation (equal), resources (equal), validation (equal), visualization (equal), writing – original draft (equal), writing – review and editing (equal). **Jeetendra Kumar Gupta:** data curation (equal), formal analysis (equal), resources (equal), validation (equal), visualization (equal), writing – original draft (equal), writing – review and editing (equal). **Varikalla Rajashakar:** formal analysis (equal), investigation (equal), validation (equal), visualization (equal), writing – review and editing (equal). **Rajeshwar Vodeti:** data curation (equal), investigation (equal), resources (equal), validation (equal), visualization (equal), writing – review and editing (equal). **Abdul Ajeed Mohathasim Billah:** data curation (equal), formal analysis (equal), resources (equal), validation (equal), visualization (equal), writing – review and editing (equal). **G. Dharmamoorthy:** data curation (equal), formal analysis (equal), investigation (equal), resources (equal), writing – review and editing (equal). **Uppuluri Varuna Naga Venkata Arjun:** data curation (equal), investigation (equal), validation (equal), visualization (equal), writing – review and editing (equal). **Voleti Vijaya Kumar:** data curation (equal), formal analysis (equal), resources (equal), writing – review and editing (equal). **Muath Suliman:** formal analysis (equal), funding acquisition (equal), validation (equal), visualization (equal), writing – review and editing (equal). **Talha Bin Emran:** conceptualization (equal), project administration (equal), supervision (equal), validation (equal), visualization (equal), writing – review and editing (equal).

## Disclosure

The authors confirm that no paper mill and no artificial intelligence were used.

## Ethics Statement

The authors have nothing to report.

## Consent

The authors have nothing to report.

## Conflicts of Interest

The authors declare no conflicts of interest.

## Data Availability

The authors have nothing to report.
